# Correction of hyperglycemia after surgery for diabetic foot infection and its association with clinical outcomes

**DOI:** 10.1186/s13104-022-06150-9

**Published:** 2022-07-27

**Authors:** Céline S. Moret, Madlaina Schöni, Felix W. A. Waibel, Elin Winkler, Angelina Grest, Bettina S. Liechti, Jan Burkhard, Dominique Holy, Martin C. Berli, Benjamin A. Lipsky, Ilker Uçkay

**Affiliations:** 1 Balgrist University Hospital, University of Zurich, Forchstrasse 340, 8008 Zurich, Switzerland; 2 Department of Medicine, University of Washington, Seattle, USA

**Keywords:** Diabetic foot infections, Surgery, Glycemia, Insulin therapy, Outcomes

## Abstract

**Objective:**

Constantly high glycemia levels might influence outcomes in the management of patients undergoing surgery for diabetic foot infections (DFI). In our center for DFI, we performed a case–control study using a multivariate Cox regression model. Patients developing a new DFI could participate in the study several times.

**Results:**

Among 1013 different DFI episodes in 586 individual adult patients (type I diabetes 148 episodes [15%], 882 [87%] with osteomyelitis; median antibiotic therapy of 21 days), professional diabetes counselling was provided by a specialized diabetes nurse in 195 episodes (19%). At admission, blood glucose levels were elevated in 110 episodes (11%). Treatments normalized glycemia on postoperative day 3 in 353 episodes (35%) and on day 7 for 321 (32%) episodes. Glycemia levels entirely normalized for 367 episodes (36%) until the end of hospitalization. Overall, treatment of DFI episodes failed in 255 of 1013 cases (25%), requiring surgical revision. By multivariate analysis, neither the provision of diabetes counseling, nor attaining normalizations of daily glycemic levels at day 3, day 7, or overall, influenced the ultimate incidence of clinical failures. Thus, the rapidity or success of achieving normoglycemia do not appear to influence the risk of treatment failure for operated DFI episodes.

## Introduction

Diabetes mellitus [1] and chronic hyperglycemia [2, 3] are known risk factors for developing most types of community-acquired and nosocomial orthopedic infections [4]. When treating diabetic foot infections (DFI), especially in patients requiring surgical procedures, the presence of persistently high blood glucose values could interfere with the host immune response against infection or compromise wound healing [2, 3]. Therefore, many experts recommend rapid correction of (postsurgical) hyperglycemia as part of the therapeutic management of DFI [4], hoping for more rapid, and ultimately more successful, control of infection. This plausible presumption has not, however, been tested and needs confirmation in a clinical setting.

In this retrospective cohort study, we assessed the association of the rapidity of normalization of glycemia to various clinical outcomes in patients undergoing operated treatment for DFI. We hypothesized that neither more rapid nor ultimately successful attainment of postoperative normoglycemia would be associated with a reduced rate of clinical failure in treatment of DFI cases. Additionally, we investigated whether there was any benefit of postoperative diabetes counselling (performed by a diabetes specialized nurse [DSN]) on the rate of clinical failure.

## Main text

### Materials and methods

#### Setting

The Balgrist University Hospital in Zurich is a tertiary referral center for patients with DFI, offering emergency service and 24-h elective consultations. with special expertise for those requiring amputations and other surgical procedures. There is a multidisciplinary team for managing DFI cases, composed of four diabetic foot surgeons, three internal medicine physicians, a hospital pharmacist, five specialized wound nurses, expert musculoskeletal radiologists, a diabetes nurse, three nutritionists, a shoemaker, a prosthesis specialist, and up to four infectious diseases physicians who are specialized in managing orthopedic infections. Moreover, this team is supported by: an in-house manufacturer of orthopedic footwear (Balgrist Tec) and other individual adaptations and devices for pressure off-loading; a re-education unit; a physical therapy service; a research unit (Balgrist Campus) with a BioBank; and, a Unit for Clinical and Applied Research with nine study nurses and two experts in investigative designs [5]. For this study, we included all episodes of DFI among adult patients treated in our center from 14 February 2000 to 31 August 2020. A patient could be included in the study more than once, as long as they developed a new DFI (after successful treatment of the index infection) on the same or contralateral foot.

We defined the presence and severity of DFI, including diabetic foot osteomyelitis (DFO), according to the International Working Group on the Diabetic Foot (IWGDF) criteria [6]. Patients were classified as having a “clinical failure” if they met any indication for needing revision surgery or resection/amputation at the same anatomical location within 1 year of treatment. For microbiological assessments, we only used results of culture of specimens of intraoperative tissue or pus, and ignored those of superficial swabs. We defined a “microbiological relapse” as a clinically recurrent DFI at the same anatomic site from which culture specimens yielded at least 50% of the same pathogen(s) as detected during the index episode of DFI or DFO. We considered “normoglycemia” as a blood glucose levels < 6.1 mmol/l (< 110 mg/dl) sustained over several days, and arbitrarily assessed three specific timepoints: at admission, and on the mornings of the postoperative days 3 and 7. Seeking standardization, we only used the results of blood glucose levels processed in our clinical laboratory, and not those obtained by nursing staff on the ward or the patient's self-measurements.

Our hospital employs one DSN (who works 40% full-time equivalence and has 9 years of professional experience) and two attending internal medicine physicians with 28- and 40-years' experience in diabetes management, respectively. Our standard practice is to aggressively treat hyperglycemia, aiming to normalize blood glucose levels as rapidly as possible. This would be accomplished either using the individual patient’s existing hypoglycemic control schemes, or by prescribing rapid-acting insulins (aspart and/or lispro).

#### Statistical analyses

Our primary outcomes of interest were the rates of “clinical failure” and “microbiological relapse” associated with attaining normoglycemia on postoperative days 3 and 7. Secondary outcomes were the relationship of DSN interventions to clinical failure, length of hospital stay, number of surgical debridement, and the prescribed duration of antibiotic therapy. We compared rates of outcomes with the Pearson-χ^2^ or the Wilcoxon-ranksum-test, adjusting for case-mix by multivariate Cox regression analysis. With over 1000 DFI episodes, the study is sufficiently powered. Because of the aleatory nature of the various types of blood glucose samples (morning versus evening, postprandial vs. fasting, on different days and frequencies, routine indication vs. demand), we did not produce standardised Kaplan–Meier curves, limited our survival assessments to the Cox regression, and did not use artificial imputing of missing data. We used STATA™ software (Version 14; College Station, USA).

### Results

We included 1013 DFI episodes that occurred during the 20-year study period in 586 individual patients (median age 67 years; 219 females [22%], type I diabetes in 148 episodes [15%], median duration of diabetes 19 years, insulin therapy in 753 [74%], median length of antibiotic therapy of 21 days), DFO in 882 [87%], revascularizations in 572 [56%] (Table 1). Among the 586 patients included in the study, 427 had at least one additional new DFI during the study period (range, 1 to 11 additional episodes). The median medical follow-up period was 7.7 years. Our DSN followed 195 episodes (19%) with a median of 1 postoperative consultation session (range, 0–8 sessions).

**Table 1:** Study population with and without clinical failures or microbiological relapse

n = 1013 DFI episodes	Clinical failure^a^	*p*-value*	Remission	*p*-value*	Microbiological relapse^a^
Male sex	203 (80%)	0.59	591 (78%)	0.43	59 (83%)
Median age	65 years	0.09	68 years	0.96	67 years
Median duration diabetes	18 years	**0.03**	20 years	0.86	18 years
Diabetic foot osteomyelitis	213 (84%)	0.06	669 (88%)	0.39	39 (83%)
Insulin therapy	192 (75%)	0.69	561 (74%)	0.51	33 (70%)
Peripheral arterial disease	559 (74%)	**0.03**	205 (80%)	0.59	37 (79%)
Revascularization	164 (64%)	**0.01**	408 (54%)	0.64	25 (53%)
Median no. debridement	1	0.10	1	0.06	1
Median duration antibiotics	30 days	**0.01**	20 days	0.18	28 days
Median glycated hemoglob	7.5%	0.16	8.3%	0.32	8.3%
Median glycemia preoperat	7.8 mmol/l	0.27	8.4 mmol/l	0.10	8.6 mmol/l
Preoperat. high glycemia^b^	55 (30%)	0.16	70 (24%)	0.12	13 (16%)
Diabetes Nurse Counselling	54 (23%)	0.34	143 (17%)	n. a	n. a
Median no. consult	1	0.38	1	n. a	n. a
Normal glycemia by Day 3	90 (80%)	0.21	262 (85%)	0.12	27 (75%)
Median glycemia by day 3	7.4 mmol/l	0.27	7.1 mmol/l	0.51	7.7 mmol/l
Normal glycemia by day 7	86 (80%)	**0.02**	235 (88%)	**0.03**	25 (74%)
Median glycemia by day 7	7.2 mmol/l	0.30	6.7 mmol/l	0.83	6.9 mmol/l
Normalized glycemia	92 (85%)	0.19	275 (90%)	0.09	28 (80%)

n. a.: not applied due to low number of events of interest in the corresponding analysis

*P-values derived from a Pearson-χ^2^ or the Wilcoxon-ranksum-test comparing the variables between the neighbouring columns. Statistically significant results are displayed in bold

^a^The denominators are not constant and rely on available values (missing data not imputated)

^b^Normal glycemia morning level set at 6.1 mmol/l (110 mg/dl)

At admission, hyperglycemia was present in 110 episodes (11%), with a median serum glucose and glycated hemoglobulin (HbA1c) values of 7.9 mmol/l and 7.6%, respectively. Glycemia normalized in 353 episodes (35%) on day 3, and in 321 episodes (32%) on day 7. After day 3 there was no further improvement in glycemia levels. Sustained normoglycemia was present in only 367 episodes (36%), starting from day 3 until the end of hospitalization.

Overall, clinical failure occurred in 255 DFI episodes (25%), of which 47 (5%) were also a microbiological relapse. In group comparisons, the presence of normoglycemia at day 3 was not associated with a lower rate of treatment failure. Among the variables we analyzed, only persistent hyperglycemia at day 7 was associated with both clinical failure and microbiological relapse (Table 1). As expected, the variables “peripheral arterial disease” and “revascularization” were associated with clinical failure, but not with microbiological relapse.

By multivariate analysis, no glycemia-related variable was statistically associated with treatment failures. The risk of adverse outcomes was not associated with HbA1c values, insulin therapy, or normoglycemia on day 3 or on day 7 (Table 2). Having had, or the number of, DNS consultations or inpatient interventions were also not associated with clinical failure (Tables 1 and 2) or a reduced need for supplementary surgical debridement (median 1 versus 1 surgery; *p* = 0.83). However, having DSN consultations was associated with a shorter hospital stay (median 14 days vs. 17 days; *p* < 0.01) and reduced duration of antibiotic therapy (19 days vs. 22 days; *p* = 0.01).

**Table 2:** Univariate and multivariate associations *(Cox regression analyses with hazard ratios and 95% confidence intervals)* targeted to the outcome “*clinical failure*”

Clinical failures, n = 255	Univariate	Multivariate
Diabetic foot osteomyelitis	1.1, 0.8–1.5	–
Symptomatic peripheral arterial disease	1.1, 0.8–1.5	–
Revascularisation	1.2, 0.9–1.5	1.1, 0.7–1.9
Number of consultations by Diabetes Nurse	***1.3, 1.1–1.4***	1.1, 0.9–1.3
Glycated hemoglobulin % at admission	1.0, 0.9–1.1	0.9, 0.8–1.1
Preoperative glycemia > 6.1 mmol/l	1.4, 0.9–2.0	2.2, 0.9–5.5
Glycemia at admission (*continuous variable*)	1.0, 0.9–1.1	1.0, 0.8–1.1
Normalized glycemia during hospitalization	0.8, 0.5–1.3	1.4, 0.6–3.2
Normalized at day 3	0.8, 0.5–1.3	1.0, 0.4–2.3
Normalized at day 7	***0.6, 0.4–1.0***	0.6, 0.3–0.3
Glycemia at day 3 (*continuous variable*)	1.1, 1.0–1.1	0.2, 0.1–2.6
Glycemia at day 7 (*continuous variable*)	1.1, 1.0–1.1	0.2, 0.1–1,4
Receiving insulin therapy	0.9, 0.7–1.2	0.8, 0.5–1.4

Statistically significant results are displayed in bold and italic

–: not included in the model due to interaction (effect modification) or lack of relevance to the study question

### Discussion

In our single-center study of adult patients who underwent operative (as well as antibiotic) treatment for a DFI, 11% had an elevated blood glucose level before their surgery. This was likely related to the presence of the foot infection. Postoperatively, only a third of all studied episodes achieved normoglycemia that remained sustained until their hospital discharge. The majority of patients left the hospital without complete normalization of their blood sugar levels.

Overall, any benefit of normalizing blood sugar levels during the postoperative hospitalization period remained insignificant. The risk of treatment failure in DFI episodes with versus without normoglycemia was essentially the same. Similarly, the rapidity of glycemic normalization did not seem be associated with the final outcomes of treatment. Likewise, the number of inpatient interventions by the DSN failed was not associated with the risk of clinical failures after surgery. In contrast, providing professional DNS counselling may have been beneficial, as it was associated with both a significantly shorter hospital stay as well as a shorter duration of overall antibiotic use. The reasons for these benefits of DNS counselling are unclear, but we speculate that patients who are able to understand and actively follow these consultations are also those with a more favorabile general outcome [4].

Our findings are not surprising for a research study investigating the management of DFI and DFO. In light of the large case-mix inherent to DFI episodes and therapies, no single variable is usually sufficient to independently determine the individual fate of the multifaceted problem of the infected diabetic foot [4, 6]. For example, in our previous investigations we found that none of the various modalities of the therapeutic interventions altered the remission incidence of (operated) DFI or DFO. These intervention variables included the duration of the antibiotic therapy [7], the number of surgical debridements undertaken [7], and the use of hyperbaric oxygen [7]. It appears that only selected chronic, patient-related parameters that are causative or contributive to the DFI, such as limb ischemia, enhanced immune suppression [8], or the anatomic localization of the infection in the foot [9], make a significant difference in multivariate analyses. Hence, our finding of a lack of a immediate benefit from achieving rapid glycemic control is consistent with the results of our prior studies. Normoglycemia seems to be less clinically important prognostically than factors such as limb ischemia, impaired immunity or patient nonadherance [4].

While studies in the intensive care setting have linked tight glycemic control [3, 10] or a low HbA1c baseline value [2] to better clinical outcomes, the scientific literature remains sparse regarding the effect of (postoperative) normoglycemia on therapeutic outcomes of DFIs. Indeed, the available literature exclusively concerns non-infected diabetic foot ulcers (DFU) [11]. For example, one prospective, observational study found that the patient's HbA1c level at baseline was not a significant predictor of ulceration, but it was a significant predictor for complete ulcer healing [12]. Two other articles suggested a possible benefit of intensified insulin treatment in DFU healing. Both stated that future randomized studies are under way in field of non-infected DFUs [13, 14]. The most recently published International Workling Ground on the Diabetic Foot guideline on infection suggests clinicians should “*Optimize glycaemic control, if necessary with insulin*,” but without going into further details [15]. There are also some published articles arguing against tight glycemic control. In one study in patients with DFU failed to find any associations of baseline HbA1c values and wound healing [16]. The available data led the Clinical Guidelines Committee of the American College of Physicians, to state that intensive glycemic control in hospitalized medical and surgical patients does not show a consistent benefit, and even shows harm [3].

## Limitation

The main strengths of our study are that it was conducted in an academic diabetic foot unit, which has a large number of patients in a database (over 1000 DFI episodes) who had a long duration of follow-up. The main limitations are: it was retrospective (making it impossibe to distinguish between medically-induced versus spontaneous normoglycemia); it had a large case-mix; the analysis was limited to the inpatient period; and, the majority of cases were operated patients (with a mixed reason for hyperglycemia due to surgery, infection, or both). In light of these limitations, we are currently performing several prospective, randomized trials investigating the duration of antibiotic in DFIs, during which we plan to assess the role of daily glycemia [5]. We hope to determine if glycemic values could be useful as a prospective predictors [5] of imminent clinical failures during DFI therapy. Lastly, in this retrospective analysis, we focused on hyperglycemia, and did not assess any possible associations of serum inflammatory markers with treatment outcomes (e.g., TNF-alpha, NF-kB or interleukins) [17].

## Data Availability

The datasets used and/or analyzed during the current study are available from the corresponding author on reasonable request.

## Abbreviations

DFI: Diabetic foot infection
DFO: Diabetic foot osteomyelitis
DFU: Diabetic foot ulcer
DSN: Diabetes specialized nurse
HbA1C: Glycated serum hemoglobulin level
ITT: Intensified insulin treatment
IWGDF: International Working Group on the Diabetic Foot
TNF-alpha: Tumor necrosis factor alpha
NK-kB: Nuclear factor-kB
